# Significantly Longer Envelope V2 Loops Are Characteristic of Heterosexually Transmitted Subtype B HIV-1 in Trinidad

**DOI:** 10.1371/journal.pone.0019995

**Published:** 2011-06-17

**Authors:** Aneisha M. Collins-Fairclough, Manhattan Charurat, Yuka Nadai, Maria Pando, Maria M. Avila, William A. Blattner, Jean K. Carr

**Affiliations:** 1 Biology Division, Faculty of Science and Sport, University of Technology, Kingston, Jamaica; 2 Institute of Human Virology, University of Maryland, Baltimore, Maryland, United States of America; 3 Centro Nacional de Referencia para el Sida, Facultad de Medicina, Universidad de Buenos Aires, Buenos Aires, Argentina; INSERM, France

## Abstract

**Background:**

In Trinidad and the wider Caribbean, subtype B Human Immunodeficiency Virus-type 1 (HIV-1B) overwhelmingly accounts for HIV infection among heterosexuals; this contrasts with the association of HIV-1B with homosexual transmission and injecting drug use globally. The HIV envelope contains genetic determinants of cell tropism and evasion from immune attack. In this study we investigate the genetic properties of the *env* V1-C4 of HIV-1B soon after transmission to Trinidadian heterosexuals. This will reveal distinctive genetic features of the strains that cause the HIV-1B epidemic in Trinidad and generate insights to better understand their properties.

**Methodology/Principal Findings:**

Quasispecies sampling was performed on the *env* V1-C4 of HIV-1B strains soon after transmission to heterosexual Trinidadians in a cohort of seroconverters. Phylogenetic relationships were determined for these quasispecies and the length and number of asparagine (N) linked glycosylation sites (NLGS) in their variable loops compared to that for HIV-1B globally. Signature amino acids within the constant domains of the *env* V1-C4 were identified for heterosexually transmitted HIV-1B from Trinidad relative to HIV-1B globally. HIV-1B obtained from Trinidadian heterosexuals soon after seroconversion had significantly longer V2 loops with one more glycosylation site, shorter V3 loops and no significant difference in V1 or V4 when compared to HIV-1B obtained soon after seroconversion from infected individuals in the rest of the world. HIV-1B soon after seroconversion and during chronic infection of Trinidadians was not significantly different, suggesting that distinctly long V2 loops are characteristic of HIV-1B in Trinidad. A threonine deletion at position 319 (T319-) along with the substitutions R315K and S440R were found to be distinctly associated with HIV-1B from Trinidad compared to HIV-1B globally.

**Conclusions:**

This finding of distinctive genetic features that are characteristic of HIV-1B strains from Trinidad is consistent with the Trinidad epidemic being established by a founder strain or closely related founder strains of HIV-1B.

## Introduction

Subtype B Human Immunodeficiency Virus-type 1 (HIV-1B) is typically associated with HIV epidemics among men who have sex with men (MSM) and injecting drug users worldwide, but in the Caribbean, HIV-1B is responsible for the heterosexual HIV-1 epidemic [1], [2]. The introduction of HIV-1 into Trinidad is proposed to have been via homosexual contact with North American men [3] and the virus later introgressed into the heterosexual population via a bisexual bridge [4]. During the mid 1980s to early 1990s, the HIV-1 epidemic in Trinidad expanded explosively within the heterosexual population and this epidemic was caused by closely related phylogenetic strains of HIV-1B [4].

The gp120 *env* of HIV-1 is essential for virus transmission, entry into host cells and immune escape. *Env* gp120 is the most variable region of the HIV-1 genome and has five constant (C1–C5) domains interspersed with five variable domains (V1–V5). Variability within *env* produces different phenotypes of HIV-1 cellular tropism and resistance to immune assault [5], [6]. Here we report molecular features within *env* V1–V4 that are distinctly associated with heterosexually transmitted HIV-1B from Trinidad. These results may also have implications for disease progression in individuals who acquire HIV-1B that is circulating in Trinidad and may be informative for the development of strategies to block HIV transmission.

## Materials and Methods

### Subjects

Peripheral blood mononuclear cells (PBMCs) were obtained from 19 Trinidadian heterosexuals within 2 months of seroconversion and 8 Argentinian MSM within 3 months of seroconversion. For 5 of the 19 HIV infected Trinidadians, PBMCs were also collected 1–2 years post infection and used as a source of viral sequences from chronic infection . Blood from HIV-infected Trinidadians were collected between 1993 and 2000 as described elsewhere [4] and PBMCs stored at a repository at Duke University Medical Centre. The demographic features of the Trinidadians were previously described [4]. PBMCs from Argentinians were collected under a study of HIV-1 incidence among a Buenos Aires cohort of MSM [7]. Institutional review boards approved both studies and each subject gave informed written consent.

### Amplification and Sequencing

DNA was extracted from PBMCs using the QiAmp DNA extraction kit (QIAgen, Valencia, CA, USA) and subjected to single genome amplification (SGA) [8], in which the template was end point diluted until no more than 40% of PCR replicates produced amplicons. The *env* V1-C4 region of HIV proviral DNA was amplified and sequenced on the Applied Biosystems 3130xl automated sequencer using Big Dye terminators (Applied Biosystems) and sequences assembled using Sequencher v4.6 (GeneCodes Corporation, Ann Arbor, MI).

### Sequence Analysis

Sequences were screened for laboratory contamination using HIV BLAST. Hypermutants were detected using HYPERMUT v2.0 (http://www.hiv.lanl.gov/content/sequence/HYPERMUT/hypermut.html) [9] and excluded from further analyses. All sequences were trimmed to the *env* V1-C4 (nts 6584–7601 relative to HXB2) region. CLUSTAL W was used for multiple sequence alignments and the alignments manually refined. Neighbour-joining phylogenetic trees were constructed and nucleotide diversity among quasispecies from each patient visualized using HIGHLIGHTER (http://www.hiv.lanl.gov/content/sequence/HIGHLIGHT/highlighter.html). Recombinants were detected using the Recombination Identification Program 3.0 (http://www.hiv.lanl.gov/content/sequence/RIP/RIP.html) and intrapatient quasispecies diversity computed using maximum composite likelihood. The variable loops V1, V2, V3 and V4 were identified in each sequence and correspond to amino acids 131–156, 157–196, 296–331 and 385–418, respectively, of the HXB2 envelope. Potential N-linked glycosylation sites (NLGS) were identified using N-GLYCOSITE (http://www.hiv.lanl.gov/content/sequence/GLYCOSITE/glycosite.html) [10] and genotypic coreceptor analysis of V3 executed using WEBPSSM (http://fortinbras.us/cgi-bin/fssm/fssm.pl) [11]. Mann–Whitney Rank Sum tests were used for comparison between groups. VESPA (http://www.hiv.lanl.gov/content/sequence/VESPA/vespa.html) was used to identify signature amino acids within C2–C3 and C4 (amino acids 197–384 and 419–459 respectively, relative to the HXB2 envelope) of recently transmitted HIV-1B from Argentina and Trinidad. Fishers exact test was used to establish whether the signature amino acids identified by VESPA were significantly associated with each group. All reported p-values are 2 sided.

### Nucleotide sequence accession numbers

Sequences generated in this study were deposited in Genbank under accession numbers HM126017-HM126454. Numerous HIV-1 subtype B sequences from the GenBank database were used for comparative analyses in this study. The GenBank accession numbers for the database sequences used are stated below. Database sequences of HIV-1 from Argentinian men who have sex with men (DQ383750, DQ383752 [16]), database sequences of HIV-1 from heterosexuals (AJ417429-AJ417431 [17]; AY308760-AY308762 [18]; AY535447-AY535454 [19]; AY835447, AY835449 [20]; DQ821488 [21]; EF593236, EF593237, EF593280, EF593282; EU588776- EU588785 [22]; EU577388-EU577403, EU576706-EU576726, EU577404-EU577424, EU577447-EU577461, EU577479-EU577509, EU577604-EU577628, EU577645-EU577674, EU576728-EU576757 [15]; FJ496167-FJ496172, FJ496174- FJ496184 [23]), database sequences of HIV-1 from men who have sex with men (AJ007943-AJ007945; DQ853427-DQ853435 [24]; EU184091-EU184096, EU184101-EU184206, EU184208-EU184217, EU184219-EU184227, EU184230-EU184241, EU184243-EU184241, EU184243-EU184244, EU184246-EU184279, EU184289-EU184291, EU184293-EU184296, EU184298-EU184413, EU184415-EU184434, EU184437-EU184451, EU184453-EU184456, EU184458-EU184465, EU184474-EU184490, EU184492, EU184501-EU184538, EU184540-EU184571, EU184573-EU184597, EU184599-EU184638, EU184642-EU184657 [25]; EU588732-EU588762, EU588833-EU588841, EU588857-EU588869, EU588897-EU588897 [22]; EU576274, EU576276-EU576292, EU576294-EU576302, EU576303-EU576321, EU576323-EU576342, EU576396-EU576424; EU576471-EU576500, EU576502-EU576553, EU577039-EU577045, EU577047-EU577060, EU577062-EU577100, EU577102-EU577118, EU577344-EU577350, EU577352-EU577364, EU577366-EU577379, EU577381-EU577387, EU577425-EU577446, EU577510-EU577552, EU577554-EU577588, EU577675-EU577691, EU577741-EU577761 [15]; FJ496081-FJ496085 [23]), database sequences of prevalent Trinidadian HIV-1 (EU839606, EU839609-10 and HM162890-HM162896 [2]) and database sequences of HIV-1 from Haiti (EU839600-EU839604 [2], U08441, U08443-U08447 [26]).

## Results

**Figure 1:**
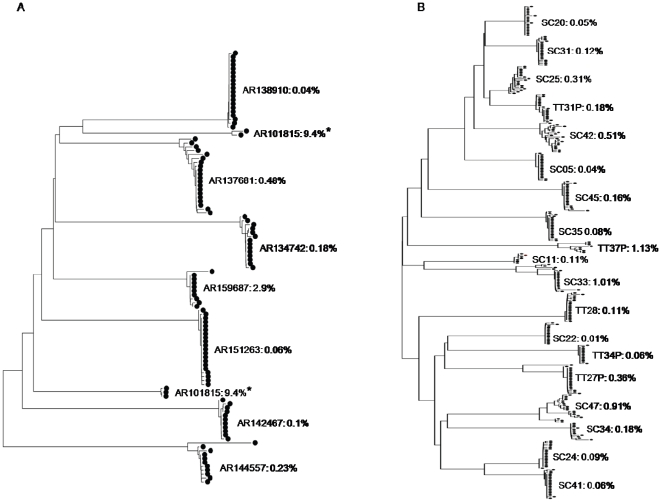
Neighbour-joining phylogeny of HIV-1 quasispecies based on *env* V1–V4. (A) Phylogeny of quasispecies isolated from Argentinean MSM and (B) Phylogeny of quasispecies isolated from Trinidadian heterosexuals. The sequences were isolated from samples collected soon after seroconversion. Percentage intrapatient diversity is presented for each patient and * highlights the patient who is infected by distantly related strains.

**Figure 2:**
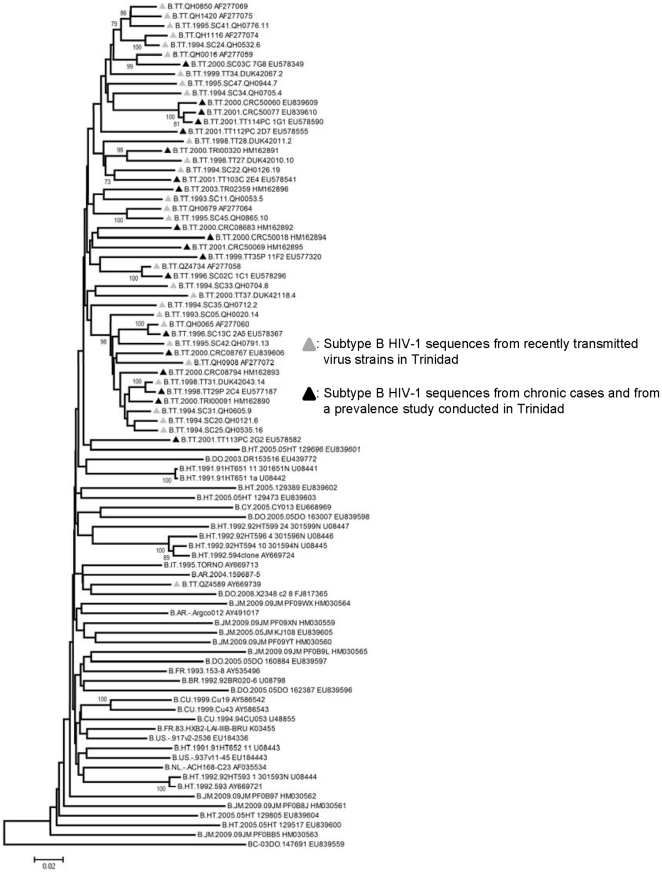
Phylogeny of subtype B HIV-1 from Trinidad. Phylogeny was inferred using the neighbour-joining algorithm and is based on *env* V1-V4.

Phylogenetic analysis confirmed that all sequences obtained were subtype B and they were all predicted to use the CCR5 coreceptor based on V3 analysis. HIGHLIGHTER and phylogenetic analysis of the quasispecies isolated indicate that a single founder variant infected 7 of the 8 Argentinians and 14 of the 19 Trinidadians, while the remaining patients were infected by two or more founder variants. Recombinant quasispecies were detected in 3 of the 5 Trinidadians who were infected by 2 or more founder variants (Table 1). The sequences from each recent seroconverter formed distinct clusters with strong bootstrap suppport (>95%) with the exception of one Argentinian patient (AR101815). The sequences from AR101815 formed two non-neighbouring clusters on the phylogenetic tree (Figure 1), suggesting that patient AR101815 was infected by two distantly related strains. Intrapatient nucleotide diversity ranged from 0.06%–9.4% (0.06%–0.48%, if AR101815 is excluded) for the recent Argentinian seroconverters and 0.04%–1.13% for the recent Trinidadian seroconverters. A phylogenetic tree was constructed using the *env* V1-C4 sequences of HIV-1B from 46 Trinidadians (19 from this study and 27 sequences from the Los Alamos Database) along with randomly choosen HIV-1B sequences from other Caribbean territories and the rest of the world. Only one representative sequence from each of the 46 Trinidadians was included in the analysis and represents all available *env* V1-C4 sequences from HIV-1B infected Trinidadian heterosexuals. With the exception of a cluster that contained 13 Trinidad HIV-1B strains and had 98% bootstrap support, the phylogenetic tree generated showed no clustering based on territory (Figure 2). The cluster of 13 Trinidad viruses consists of 10 acute sequences and 3 prevalent sequences. Of the ten acute viruses 7 were previously reported to form a subcluster within Trinidad viruses [4].

**Table 1:** Features of subtype B HIV-1 quasispecies isolated from Argentinian men who have sex with men and Trinidadian heterosexuals recently following seroconversion.

Country	Risk Factor	Patient/Sample ID	DPS	Number of Single Genomes	Number of Founder Virus(es)	Recombinants
Argentina	MSM	AR145447	90	11	1	No
Argentina	MSM	AR151263	90	20	1	No
Argentina	MSM	AR101815	75	5	2	No
Argentina	MSM	AR134742	90	14	1	No
Argentina	MSM	AR137681	90	20	1	No
Argentina	MSM	AR138910	60	20	1	No
Argentina	MSM	AR142467	90	11	1	No
Argentina	MSM	AR159687	90	10	1	No
Trinidad	Heterosexual	SC05.QH0020	10	19	1	No
Trinidad	Heterosexual	SC11.QH0053	37	7	1	No
Trinidad	Heterosexual	SC20.QH0121	26	20	1	No
Trinidad	Heterosexual	SC22.QH0126	35	15	1	No
Trinidad	Heterosexual	SC24.QH0532	34	20	1	No
Trinidad	Heterosexual	SC25.QH0535	59	20	1	No
Trinidad	Heterosexual	SC31.QH0605	25	20	1	No
Trinidad	Heterosexual	SC33.QH0704	43	20	2	Yes
Trinidad	Heterosexual	SC34.QH0705	6	12	1	No
Trinidad	Heterosexual	SC35.QH0712	34	20	1	No
Trinidad	Heterosexual	SC41.QH0776	40	20	1	No
Trinidad	Heterosexual	SC42.QH0791	7	20	≥2	Yes
Trinidad	Heterosexual	SC45.QH0865	46	20	1	No
Trinidad	Heterosexual	SC47.QH0944	30	20	2	Yes
Trinidad	Heterosexual	TT27.DUK42010	5	20	2	No
Trinidad	Heterosexual	TT28.DUK42011	3	20	1	No
Trinidad	Heterosexual	TT31.DUK42043	5	20	1	No
Trinidad	Heterosexual	TT34.DUK42067	13	13	1	No
Trinidad	Heterosexual	TT37.DUK42118	0	8	3	No

DPS = days post seroconversion.

**Figure 3:**
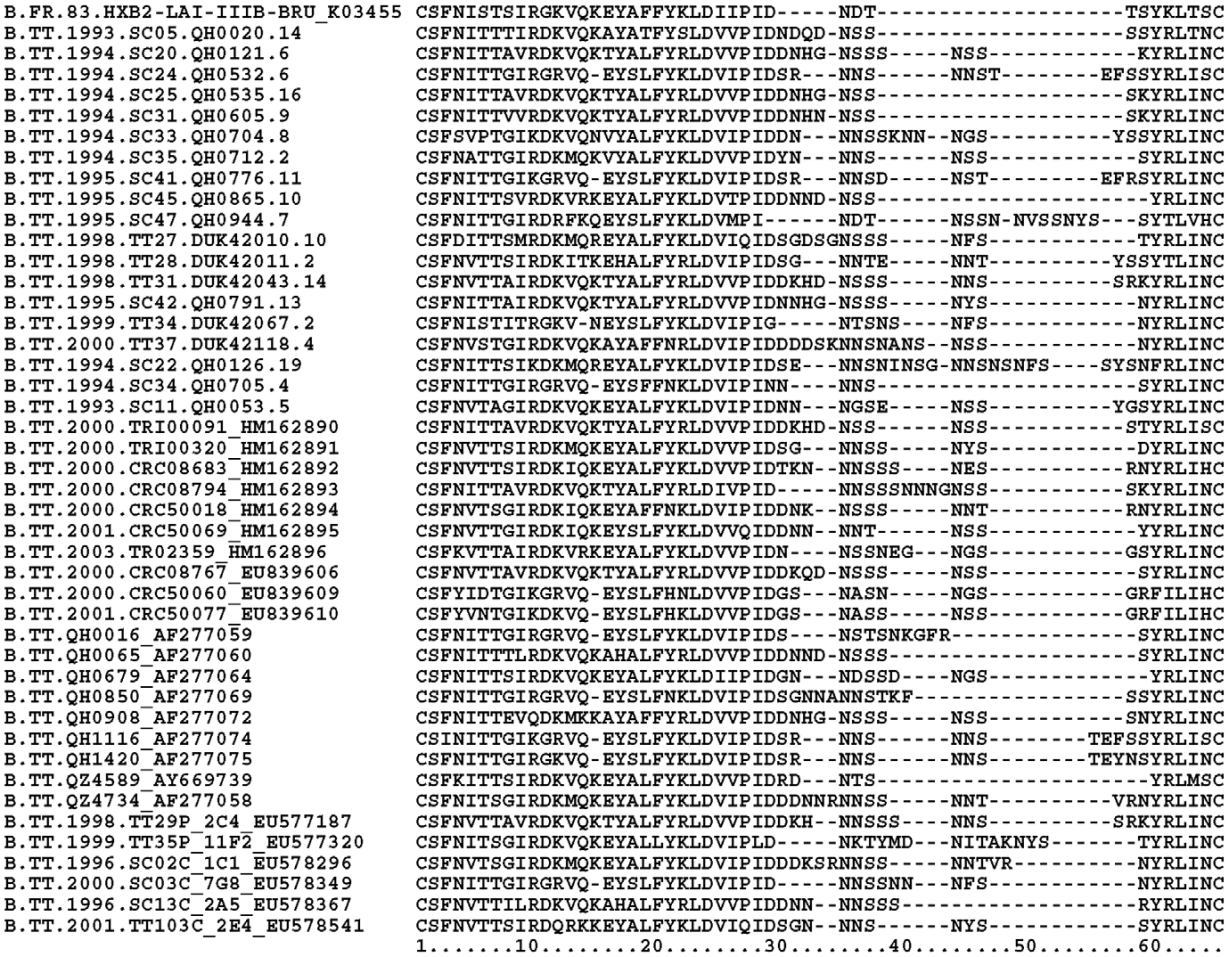
Multiple alignment of *env* V2. All available (n = 46) V2 loop sequences from subtype B HIV-1 from Trinidad are present in the alignment.

The length and number of NLGS within the contiguous V1–V4 and the individual variable loops were compared between HIV-1B obtained from Trinidadian heterosexuals soon after seroconversion, HIV-1B obtained from Argentinian MSM soon after seroconversion and database HIV-1B sequences obtained from MSM and heterosexuals by SGA within 3 months of seroconversion. The only difference observed between the groups of HIV-1B compared, was that the V2 loops of HIV-1B obtained from Trinidadian heterosexuals soon after seroconversion were significantly longer, with one more glycosylation site (Figure 3), and their V3 loops significantly shorter compared to all other HIV-1B (Table 2). There was no significant difference in the variable loops betweenHIV-1B obtained soon after seroconversion and during chronic infection from 9 Trinidadian heterosexuals or between HIV-1 obtained from Trinidadian heterosexuals soon after seroconversion and HIV-1B from a prevalence study conducted in 2000–2003 in Trinidad [2]. HIV-1B from Trinidad also had significantly longer V2 and shorter V3 than HIV-1B from Haiti (Table 2), which is believed to have the oldest HIV epidemic in the Western Hemisphere [12].

**Table 2:** Comparison of the median length and number of N-Linked Glycosylation Sites in the *Env* V2 and V3 of Subtype B HIV-1 from Trinidad and the Rest of the World (p-values represent comparison between recently transmitted HIV-1 from Trinidadian heterosexuals and others).

	V2		V3	
	Length/aa	Number NLGS	Length/aa	Number NLGS
Recently Transmitted HIV-1 from Trinidadian Heterosexuals, n = 19	46	3	34Due to T319_	1
**VS**				
Recently Transmitted HIV-1 from Argentinian MSM, n = 10*	39*P*<0.001	2*P*<0.01	35*P*<0.001	1ns
Recently Transmitted HIV-1 from Database MSM, n = 62	39*P*<0.001	2*P*<0.001	35*P*<0.001	1ns
Recently Transmitted HIV-1 from Database Heterosexuals, n = 11	39*P*<0.01	2*P*<0.01	35*P*<0.01	1ns
Chronic HIV-1 from Trinidadian Heterosexuals, n = 9	46ns	3ns	34ns	1ns
Prevalent HIV-1 from Trinidad**,n = 10	46ns	3ns	34ns	1ns
HIV-1 from Haiti, n = 11	43*P*<0.05	2ns	35*P*<0.05	1ns

ns =  not significant,

*bulk database sequences for two Argentinians within 3 months of seroconversion were included in the analyses,

**bulk database sequences [2].

Twelve amino acid positions in V1-V4 were identified by VESPA as differentiating HIV-1B that were obtained from Trinidadian heterosexuals and Argentinian MSM soon after seroconversion, however biological data is available for only three of these positions R315K, S440R and T319-. R315K and T319A (T319- was observed for the Trinidadians) in conjunction with an additional NLGS confers escape from neutralization by the anti-V3 monoclonal antibody KD-247 [13], whilst S440R is associated with resistance against candidate HIV-1 fusion inhibitor BMS378806 [14]. R315K and T319- are present in 42% and 74% of HIV-1B obtained from Trinidadian heterosexuals soon after seroconversion respectively, but are absent from HIV-1B obtained from Argentinian MSM soon after seroconversion and are present in <18% of HIV-1B database sequences obtained from heterosexual or MSM soon after seroconversion in the rest of the world. S440R is present in 90% of HIV-1B obtained from Trinidadian heterosexuals soon after seroconversion, but in <40% of HIV-1B obtained from Argentinian MSM soon after seroconversion and <60% of database sequences obtained from heterosexual or MSM soon after seroconversion in the rest of the world.

## Discussion

In this study we investigated genetic features of the *env* V1-C4 of heterosexually transmitted HIV-1B from Trinidad, to unveil whether distinct genetic features are associated with HIV-1B strains in this heterosexual epidemic and garner insights to improve our understanding of these HIV-1B strains. The majority of patients were infected by a single strain (88% Argentinians and 74% Trinidadians) and this is consistent with a low number of viruses establishing infection in sexual HIV-1 transmission [8], [15] . Half of the 20 HIV-1B infected MSM studied by Keele et al were infected by more than one virus strains [15] however we found multiple infecting virus strains in only 12% (1/8) Argentinian MSM. Compared to the study by Keele et al, we studied a smaller number of MSM and sampled fewer virus isolates from each patient. It is unclear whether our finding that a higher percentage of Argentinian MSM was infected by a single strain compared to MSM in other studies is due to our sampling limitations or natural variation between populations.

This study defines genetic features in the V1-C4 region of *env* that distinguishes heterosexually transmitted HIV-1B in Trinidad from HIV-1B transmitted globally and is consistent with the previous suggestion that the HIV epidemic in Trinidad likely resulted from a single introduction of HIV-1 [4]. Longer, more glycosylated V2 loops, shorter V3 loops (due to T319- deletion) and R315K within V3 were among the molecular signatures of heterosexually transmitted HIV-1B from Trinidad. These signatures correlate with neutralization resistance mutations developed by prototype laboratory strains in different *in vitro* studies [6], [13], [15]. Further studies to assay the neutralization activity of these virus strains must therefore be done as this has implications for disease progression and the transmission of HIV-1 among Trinidadians.
